# Consequences of repetitive head trauma: a historical reappraisal of Hobey Baker

**DOI:** 10.3389/fneur.2026.1842231

**Published:** 2026-08-19

**Authors:** Grace C. Plassche, Xavier E. Ferrer, Alan W. Reynolds, Cole R. Morrissette, Thomas A. Fortney, Michael K. Popkin, Charles A. Popkin

**Affiliations:** 1 Department of Orthopedic Surgery, Columbia University, New York, NY, United States; 2 Department of Orthopedic Surgery, Wake Forest University, Winston-Salem, NC, United States; 3 New Hampshire Orthopedic Center, Bedford, NH, United States; 4 Department of Psychiatry, University of Minnesota, Minneapolis, MN, United States

**Keywords:** Hobey Baker, repetitive head trauma, ice hockey injuries, college football injuries, traumatic encephalopathy syndrome, traumatic encephalopathy

## Abstract

**Background:**

Hobart “Hobey” Baker (1892–1918) is one of the most celebrated amateur athletes in American history. The cause of his death in a plane crash at the end of WWI has been the subject of a longstanding debate. Contemporary scientific literature links repetitive head impacts (RHIs) with neurobehavioral dysregulation and impaired judgment.

**Hypothesis:**

Hobey Baker’s exposure to repetitive head trauma impaired his judgment preceding and during his fatal final flight.

**Design, setting, and participants:**

This historical narrative analyzed biographical accounts of Baker’s life, supplemented by contextual historical media sources. A parallel literature review was conducted using PubMed to identify contemporary evidence regarding RHIs, chronic traumatic encephalopathy (CTE), and traumatic encephalopathy syndrome (TES) in contact athletes. The primary focus of this analysis was the historical record of Baker’s exposure to head trauma, symptoms, and behaviors over time.

**Main outcome(s) and measure(s):**

The primary outcome of this analysis was to review the existing literature on Hobey Baker, along with TES and CTE, in order to evaluate whether RHI contributed to Baker’s untimely death.

**Results:**

Reports of Baker’s numerous and severe head injuries, during a time when protective headgear was not common, indicate that he likely had sufficient RHI to cause significant damage to his brain. His slow descent from a kind, humble gentleman to a paranoid, irritable risk-taker illustrates the clinical manifestations of CTE, similar to those seen in modern-day athletes.

**Conclusion and relevance:**

Baker remains immortalized as the archetype of the American amateur athlete—skilled, humble, and courageous. However, the same fearlessness that defined his athletic greatness exposed him to repeated head trauma in an era devoid of protective equipment or concussion awareness. Modern neuroscience suggests that repetitive head trauma can produce neurobehavioral changes, including impulsivity, mood instability, apathy, and impaired judgment. Baker’s documented exposure to head trauma, personality evolution, and fatal decision-making align with these patterns. We assert that the circumstances leading to his death were likely a result of the head trauma he experienced throughout his life.

## Introduction

“In a desperate act, [his opponent] pulled Hobey’s skates out from under him and sent the star sliding into the iron post of the goal… Baker lay motionless, and many of the spectators feared that he had broken his neck” (1).

“Frequently called upon to spring into the immovable wall of lineman, more than any other player, only to be body slammed down with cartoon violence” (1–3).

**Figure 1:**
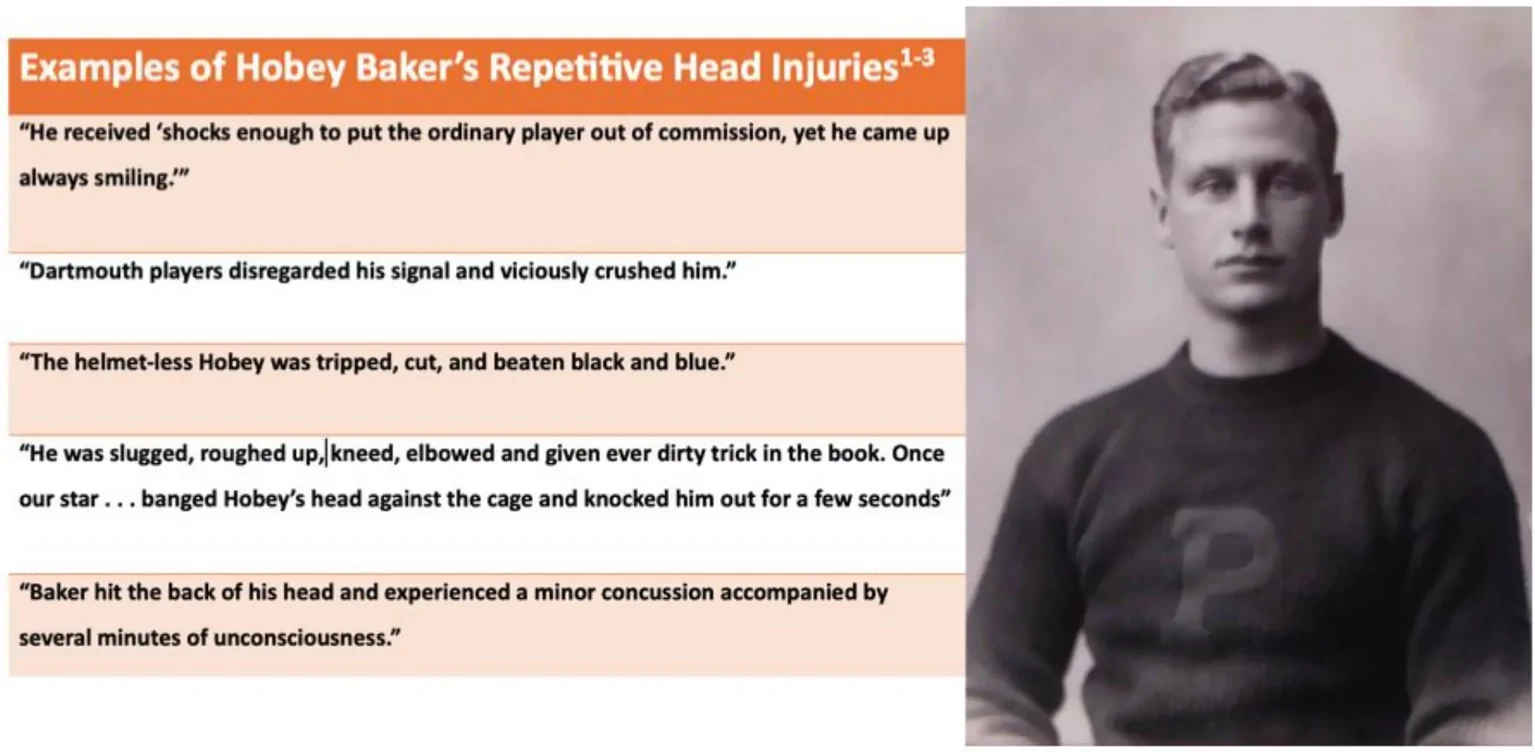
Hobart Amory Hare Baker—uploaded photo circa 1910–1914 (108th wing photo available courtesy of Princeton Frist Campus Center).

These descriptions recount the athletic career of Hobart “Hobey” Amory Hare Baker (1892–1918)—a college hockey icon, All-American football player, World War I aviator, and an enduring symbol of amateur sportsmanship. Baker remains the only athlete to be inducted into both the College Hockey Hall of Fame and the College Football Hall of Fame. Each year, the Hobey Baker Award is given to the most outstanding NCAA men’s ice hockey player, celebrating excellence, sportsmanship, and character in his honor. His death at age 26 in a plane crash shortly after World War I has been debated for over a century, with speculation ranging from mechanical failure of the plane to intentional self-harm (Figure 1).

Contemporary descriptions of Baker’s athletic career include repeated head trauma, episodes of transient loss of consciousness, and persistent exposure to violent contact in both football and hockey. Current research on repetitive head impacts (RHIs) has associated them with long-term neuropsychiatric sequelae, including impulsivity, mood swings, depression, risk-taking behavior, and impaired executive function (4, 5). This historical analysis examines whether Baker’s documented exposure to repetitive head trauma and his subsequent behavioral changes align with contemporary criteria for traumatic encephalopathy syndrome (TES). Since TES is a clinical research syndrome that does not invariably correspond to pathologically confirmed chronic traumatic encephalopathy (CTE), this study does not seek to establish a retrospective diagnosis of CTE, but rather to assess the extent to which Baker’s documented history aligns with current TES criteria.

## Methods

Primary sources related to Hobey Baker were obtained from three major biographical works by Davies, Rappleye, and Salvini (1–3). ESPN’s 30 for 30 Podcast, “The Natural,” detailing Hobey Baker’s life, supplemented these primary sources (6). A structured literature review was conducted using PubMed with search terms such as “traumatic brain injury,” “repetitive head impacts,” “chronic traumatic encephalopathy,” and “traumatic encephalopathy syndrome,” targeting athletes in contact sports.

## The making of an American hero

**Figure 2:**
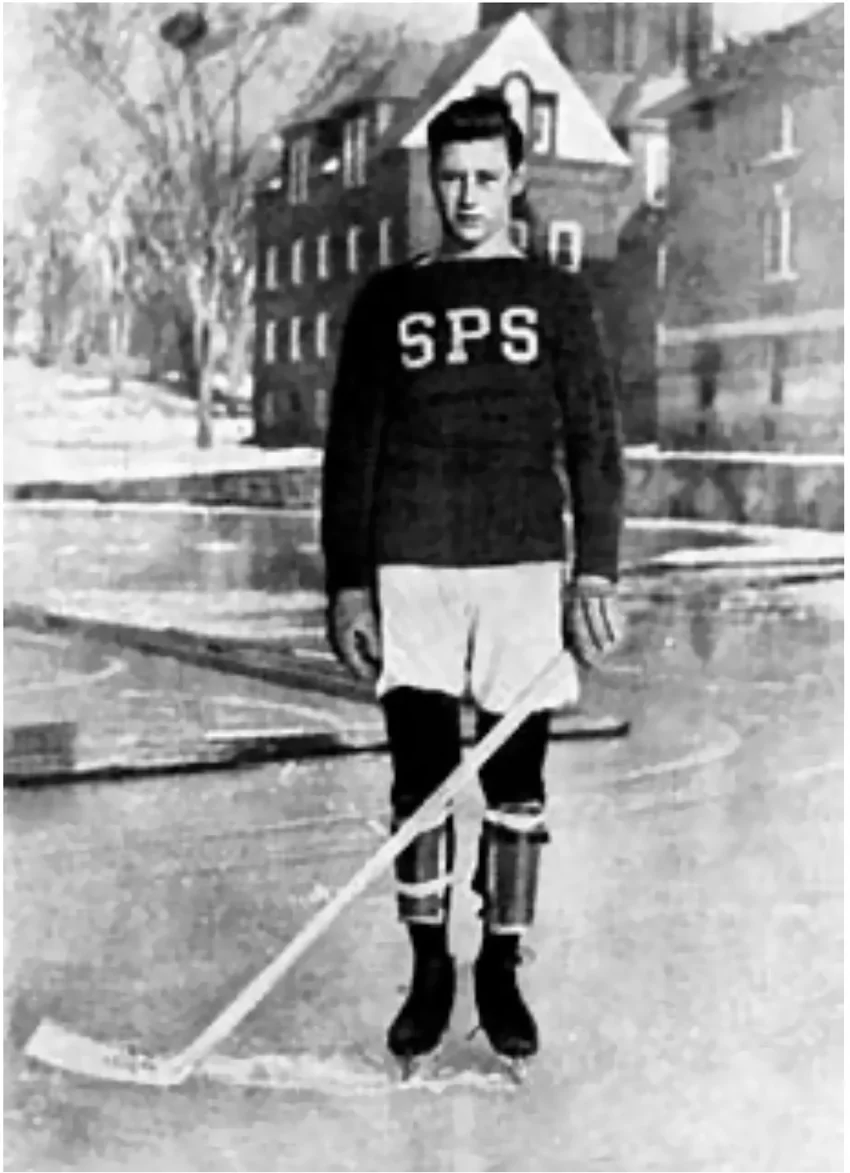
Image of Hobey Baker from his time at the St. Paul School (1908–1910 on Wikimedia Commons, uploaded from the U.S. Hockey Hall of Fame Website).

Hobey Baker was born on 15 January 1892 into a prominent Philadelphia family. He grew up skating on the frozen Wissahickon Creek with his older brother, Thornton. By 1900, Hobey’s parents’ marriage was in a state of “mutual dereliction” (2). To spare Hobey and his brother from the strife of their parents’ divorce, they were sent to St. Paul’s School, where Hobey found structure, discipline, and valuable mentorship from his father’s first cousin, James Conover. Conover was a master at the school and a hockey missionary of sorts (3). Having traveled to Canada, Conover brought the game back to the preparatory school, earning St. Paul’s the moniker the “Cradle of American Hockey” (1). Baker refined his skating ability to near-mythical levels (Figure 2). At St. Paul’s, Hobey developed into a gifted athlete, excelling in football, crew, and ice hockey. Accounts of his time at St. Paul’s portray him as reserved, courteous, and humble, valuing sportsmanship over personal acclaim (2).

Even during adolescence, Baker’s playing style involved significant physical exposure to head trauma. Protective equipment was minimal, and checking was violent. His willingness to endure punishment on the ice and gridiron became part of his legend.

### Princeton football–violence without helmets

**Figure 3:**
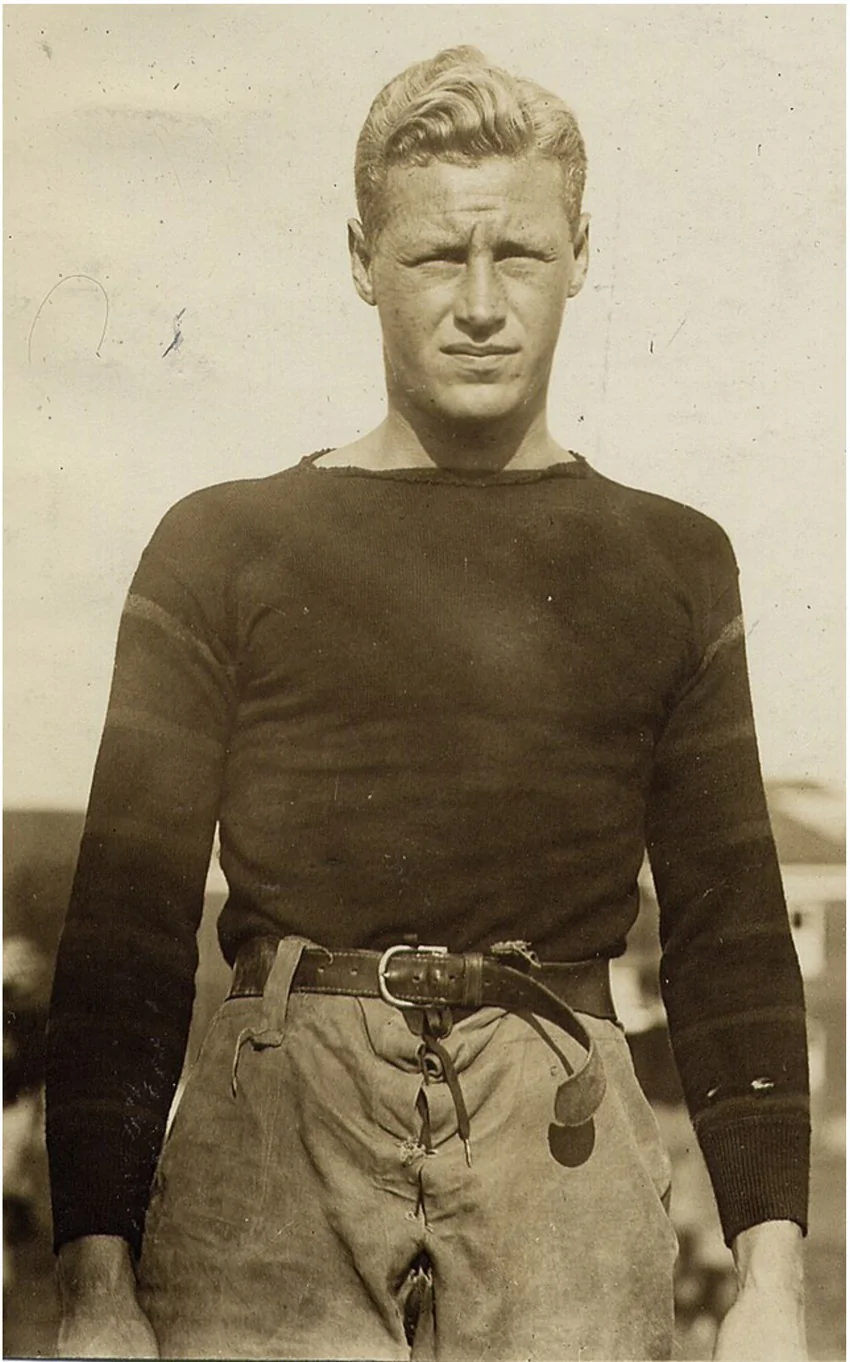
Image of Hobey Baker on the gridiron at Princeton University without a helmet (image from Wikimedia Commons, uploaded November 2010) (https://commons.wikimedia.org/wiki/File:Hobey_Baker_Princeton_Football.jpg).

Entering Princeton in 1910, Baker starred as a left halfback. Two details consistently appear in biographical accounts:

1. Baker never wore a helmet and used minimal padding (Figure 3)
2. He absorbed repeated high-impact tackles without hesitation.

**Figure 4:**
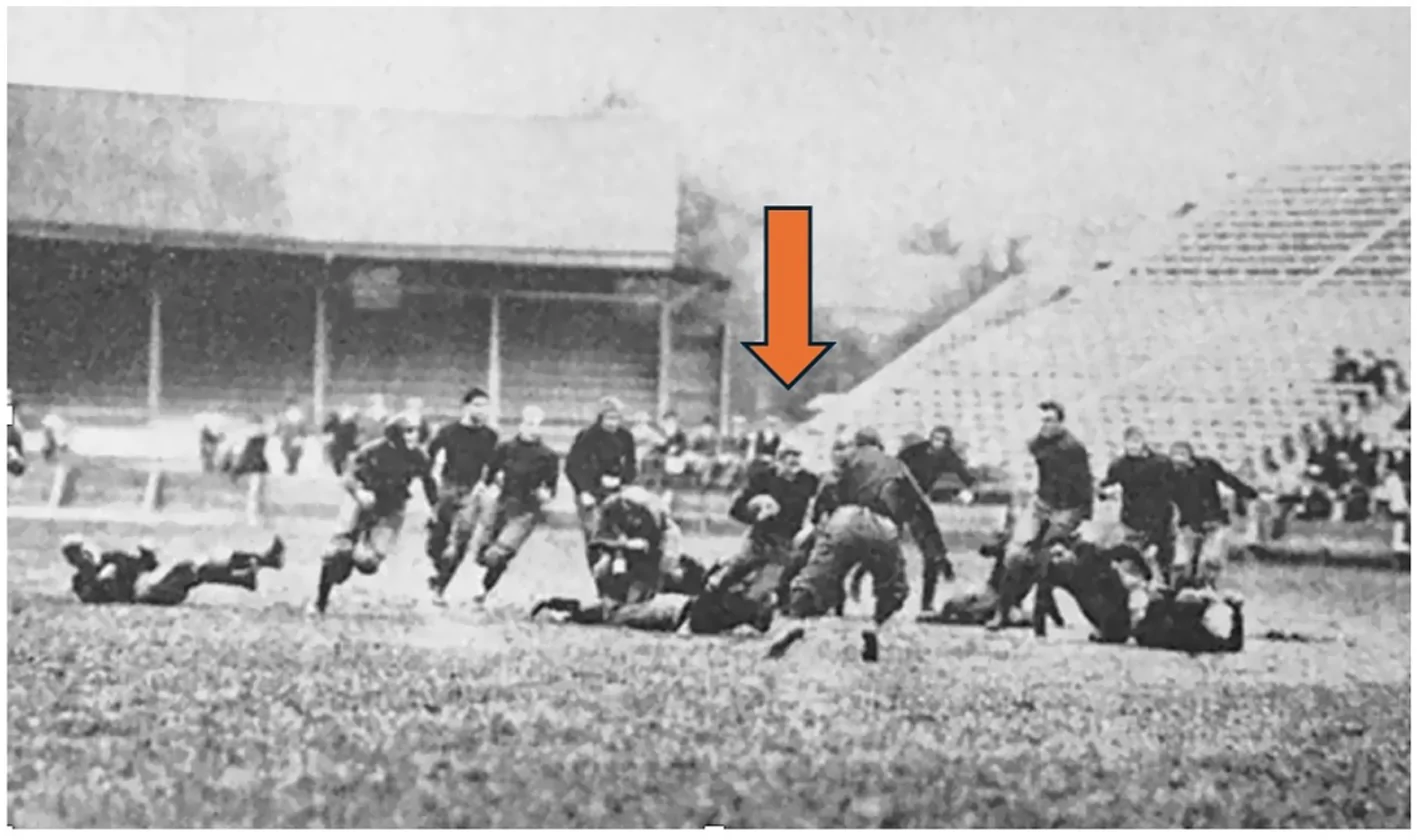
Hobey Baker making a long punt return against Syracuse, 1913. Princeton Alumni Weekly 1966.

The brutality of early 20th-century football has been well documented (2). In 1909 alone, dozens of deaths were attributed to football-related injuries (2, 7). President Theodore Roosevelt intervened to reform the sport, leading to the creation of the forward pass (8, 9). Despite these reforms, substantial violence remained prevalent in the game. Reports describe Baker routinely launching himself into defensive lines, enduring bone-crushing tackles, and repeatedly striking the ground headfirst (2) (Figure 4). In the 1911 national championship game against Yale, Hobey carried Princeton to victory, with spectators noting “why he (Hobey) wasn’t killed, (we) do not know”. His physique—a slender build with a narrow neck has been associated with high concussion risk in modern analyses (10). Stories of Hobey Baker playing football, being viciously tackled, and continuing to play conjure images of the repetitive head traumas he likely endured in an age when concussion awareness did not exist. His football career as a Tiger culminated in a single-season scoring record that stood for 50 years. Hobey caught more than 900 punts and averaged over 300 yards of offense per game (1).

### Princeton hockey: Get Baker

Hobey’s many years of skating on the ponds of New Hampshire at night, in complete darkness, rendered him a magician on ice. His opponents even commented that his skating was an “unparalleled feat of magic artistry” (1). His dominance on the ice was magnified by the elevated status of amateur sports at the time. It was considered unsportsmanlike to accept money for sports (11). During this time, it was the college athlete who garnered the praise and admiration of the public, casting a spotlight on Hobey Baker’s years as a Princeton ice hockey star.

If football exposed Baker to violence, hockey only amplified it. Hobey dealt with savagery on the ice, with physical confrontations being commonplace. As he refused to wear a helmet or to admit defeat or pain, he continued playing and accumulating injuries and repetitive hits to the head (1, 2). Opponents adopted a strategy that could be summarized with one simple phrase: “Get Baker”. He was tripped, elbowed, kneed, and struck repeatedly. Baker’s dominance on the ice made him a target, and there are numerous descriptions of repeated blows to the head, facial lacerations, and loss of consciousness (3, 6).

**Figure 5:**
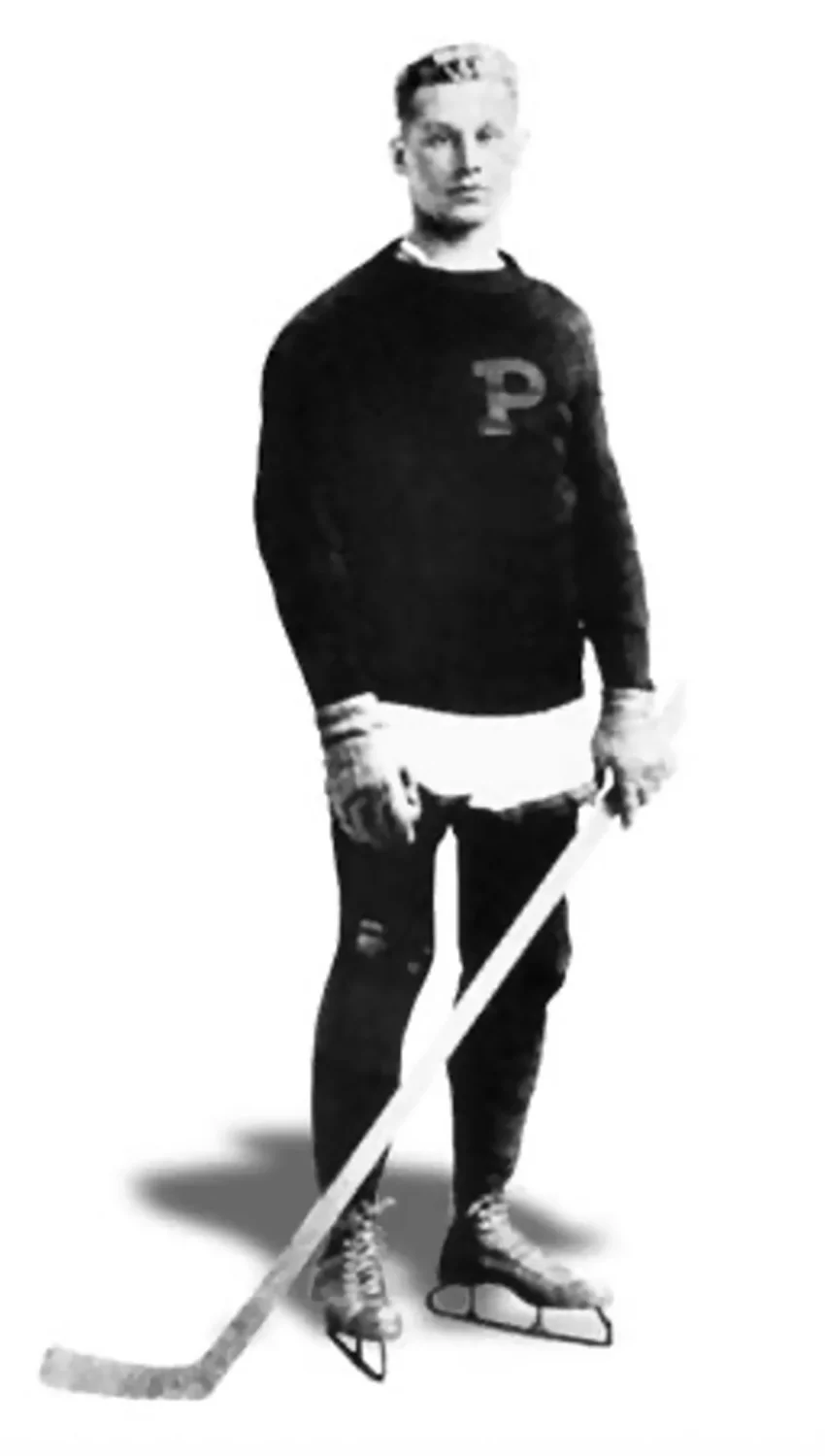
Hobey Baker image from his time with Princeton Hockey (Wikipedia Commons uploaded from the Hockey Hall of Fame website: https://www.hhof.com/HonouredMembers/MemberDetails.html?type=Player&mem=P194502&list=ByName#photo).

Despite the beatings and repetitive head trauma, Hobey’s collegiate hockey career at Princeton was extraordinary (Figure 5). He averaged approximately three goals and three assists per game and won two national championships. However, as his fame grew, subtle changes started to appear in his behavior.

### Personality shifts

For the majority of his time at Princeton, he maintained the persona he had developed at St. Paul’s. In the midst of hazing rituals, the freshmen remembered Hobey in contrast to the rest as a “friendly, courteous boy… always the gentleman, never took advantage,” and he was recognized for his presence beyond his athletic prowess, for his character, as “the idol, the positive influence for good” (1). Perhaps the most effusive examples of his character came from F. Scott Fitzgerald, the American novelist, who was in awe of Hobey, even describing him as “an ideal worthy of everything in my enthusiastic admiration” (12). The earliest descriptions of Baker portray him as a gentleman sportsman who was “almost apologetic each time he would strip the other player of a puck” (3).

Later in his years at Princeton, there were reports of reckless high-speed car accidents, dangerous stunts on the rooftops of Manhattan, and social withdrawal (3).

After graduation, Baker entered finance but appeared restless and disillusioned. He began to smoke cigarettes heavily, several accounts of him from that time highlighting his lack of zeal (6). He lamented that his life’s peak had already passed: “I realize that my life is finished… no matter how long I live, I will never equal the excitement of playing on the football fields” (3).

He continued to play post-collegiate hockey for the St. Nicholas Hockey Club (St. Nick’s) in a newly formed amateur hockey league. The competition in this postgraduate hockey league was far more physical, as several Canadians were secretly paid to play on these supposedly amateur teams (1). Players no longer harbored the gentlemanly ideals of collegiate sports and focused instead on “winning at all costs” (3). In one game, Baker received such a fierce beating that a fan in the stands shouted, “Why do not you get a club and finish the task quickly?” (3). Later that same season, Baker suffered a severe concussion after being tripped and striking the ice with his head so forcefully that he was unconscious for “several minutes” (3). The relentless assaults perhaps reached a pinnacle during this final act of his athletic career. They wore down even the resilient spirit of Hobey Baker and, by 1917, he had played his final hockey game.

In retrospect, these years mark a behavioral inflection point characterized by impulsivity, mood fluctuations, apathy, and increased risk-taking.

### Aviation and escalating risk

As the disillusionment with his life in New York reached a fever pitch, Baker threw himself into a more daring and dangerous version of his athletic endeavors. During his time as a banker, Hobey Baker enrolled in aviation courses. He began seeking out thrills and spectacles such as flying over a Princeton football game (12). Previously, he had deferred all credit to his team, and despite being terrified of “being considered cocky,” Rappleye notes in his biography that this post-graduate Baker was also “equally afraid of being ignored… his death-defying feats inevitably demanded everyone’s full attention” (3). The fear of becoming obsolete, along with increased impulsivity, defined this era of his life.

Hobey left the New York finance world and enlisted as a pilot in the World War in France. This finally satisfied Hobey’s sense of “noblesse oblige”—the idea that a privileged young man has a moral duty to serve, lead, and even die for his country (2, 13). Due to poor logistics, the majority of American pilots did not engage in combat for nearly a year after arriving in France. Hobey Baker and his squadron continued to train, which often proved to be just as deadly as the front line (3). In his letters to his old roommate Percy Pyne, he recounted a near-fatal free-fall during this period. His tone rang of apathy as he wrote: “I thought I was about to be killed, and it did not seem to worry me particularly” (3). Frustrated and almost listless due to the lack of action, Baker impulsively turned to reckless stunts, nearly causing a friend to crash after flying too close to him and then flying straight into an observation balloon after forgoing goggles (3). His account of the latter incident to his father also conjured images of his prior head trauma, as he stated: “the machine [was going] between 60 to 70 miles per hour when I struck, and turned completely over on its back, yet all I got was a large bump on my head, which knocked me silly” (3, 14).

During this time, Hobey also started a romance with Mimi Scott, a woman from a wealthy background who worked on the front lines of the war as a nurse with the Red Cross (2). Optimistic about the prospects of this relationship, he also received the news of his ever-anticipated assignment to the Lafayette Escadrille, under the command of his friend and fellow Princeton Tiger, Charles Biddle. His letters to his father during this time were filled with manic joy as he described his airborne battles and prospective kills (14). However, these sentiments were countered by his self-doubts about his love life, as he believed himself unworthy of Mimi from a financial perspective. Rappleye describes his emotional ups and downs during this time, fluctuating from “fatalistic to full-on depression” (3). Amidst this chaos and dissatisfaction, Baker and Scott made a pact of marriage if they were both to survive the war, a somewhat impulsive decision as Baker continued to question the plausibility of their relationship. The volatility of his emotions, motivations, and satisfaction with his circumstances was documented in his letters home (14). The joy of his engagement quickly soured as he realized how little contact he had with Mimi, and it was also revealed that she was being courted by the son of a wealthy diplomat in Paris. Their broken engagement was reported in a newspaper on 26 November 1918, with the headline “Dissolved by Mutual Consent of Athletic Star and Fiancée, Miss Mimi Scott” (1).

As he dealt with this heartbreak and with the war nearing its end, Baker was promoted to captain. This promotion marked the beginning of his most prolific period of aerial action and airborne battles. While shooting down German planes, “any semblance of Hobey’s sensitive empathy was long forgotten at this final stage in the war” (3).

### Final flight

He received orders to return home on 21 December 1918, after the end of WWI, with his former preceptor and assistant football coach, Donald “Heff” Herring, at his side (12). It was Herring who provided insight into the final events that led up to Hobey Baker’s death; [Herring] sat back and “watched Captain Baker parade through the room with manic energy” (3). In a show of atypical inelegance, his lack of an appropriate outlet for this frenetic energy resulted in a spur-of-the-moment decision to “take one last flight”. Furthermore, as if his decision-making were impaired, Baker opted to fly a recently repaired SPAD XIII, rather than his own plane.

**Figure 6:**
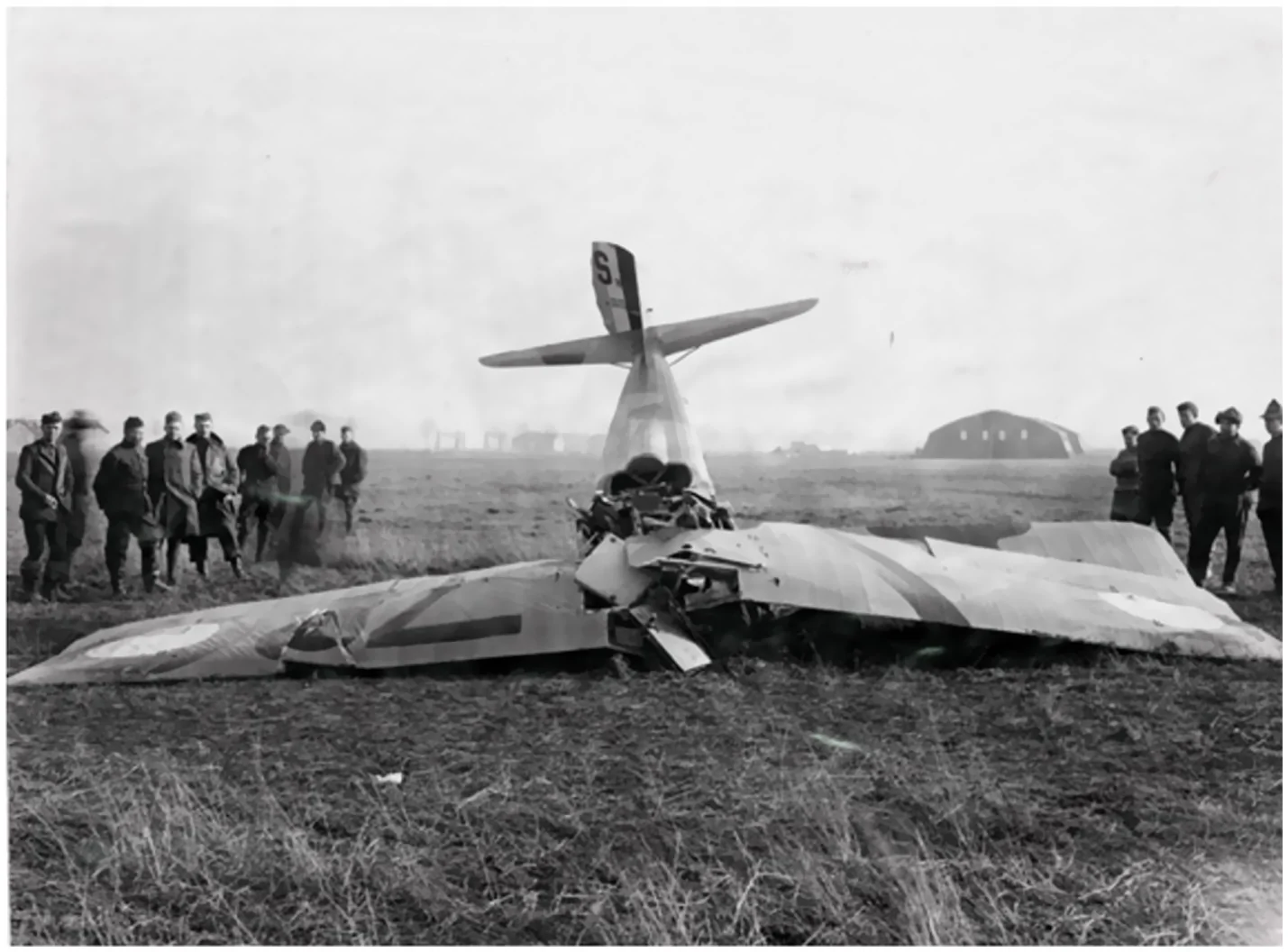
Plane crash Hobey Baker’s crashed plane. HOPD—Photo of Hobey Baker’s crashed SPAD XIII, that he was testing on the day of his intended return to the U.S. (108th wing provides access to this public domain photo available from the Princeton Library, John D. Davies Collection on Hobey Baker (AC005), box 5, folder 7, image no. 21828zv).

Multiple errors in judgment culminated in Hobey Baker impulsively taking an unnecessary flight in a plane he did not know, in a mindset wholly unprepared for what would happen. When the engine failed, instead of executing a standard glide landing, he attempted to “turn back to the field,” a cardinal sin for any pilot, especially one considered the premier aviator of his squadron. In an effort to save the plane, he tried to circle back to land on the airfield. This maneuver resulted in a loss of air speed and control, leading to the fatal crash (Figure 6). Baker had handled similar engine failures successfully; so why, on this day, did his judgment fail?

Biographers have debated accident versus suicide (1–3). However, neither framework fully explains the cascade of impaired decisions: unnecessary flight, unfamiliar aircraft, suboptimal conditions, and hazardous maneuver. Modern medicine and neuroscience offer another possibility: impaired executive function and risk assessment due to cumulative RHIs.

### Repetitive head trauma in athletes

*Dementia pugilistica*, previously known as “punch drunk” syndrome, was not formally recognized until 1928—a decade after Hobey Baker died. It was initially identified in boxers exposed to repetitive neurotrauma. This clinical syndrome, now associated with chronic traumatic encephalopathy (CTE), consists of dysarthria, ataxia, memory impairment, mood swings, and personality changes (15). In the current athletic and scientific landscape, the link between repetitive head trauma and symptoms such as impulsive behavior, depression, and poor judgment is increasingly well documented (5). The landmark 2005 neuropathological report of NFL player Mike Webster demonstrated the presence of CTE pathology associated with premortem cognitive impairment, mood disorders, and behavioral dysregulation (16).

Subsequent studies have revealed increased rates of neurodegenerative disease and suicide among professional athletes exposed to RHIs (5, 13–17). Perhaps even more alarming than these findings in professional athletes in high-contact sports are the recent findings published involving younger athletes. A recent JAMA study identified CTE pathology in athletes under 30 years of age, with many exhibiting depression, impulsivity, and suicidal thoughts (17). In addition, experimental models have demonstrated neuronal loss and neuroinflammation following repetitive impacts, even before the classic signs of CTE pathology emerge (18).

Since CTE is a postmortem diagnosis, the National Institute of Neurological Disorders and Stroke (NINDS) introduced criteria for traumatic encephalopathy syndrome (TES) (19). TES provides a framework to characterize the clinical manifestations of repetitive head trauma during an individual’s life. The criteria include: (1) exposure to repetitive head impacts, (2) cognitive impairment in episodic memory or executive functioning and/or neurobehavioral dysregulation, (3) a progressive course, and (4) the absence of another explanation for the clinical features (19). The clinical features that are highlighted in this consensus and other analyses of the clinical presentation of CTE include impulsive behavior, paranoia, apathy, memory problems, motor problems, and impaired judgment (20). It has been universally shown that the effects of repetitive head impacts (RHIs) are multiplicative, with an increase in RHIs decreasing the threshold for subsequent concussions and increasing the risk of experiencing symptoms (21).

### Applying TES criteria to Hobey Baker

Hobey Baker’s life demonstrates the following:

1. Extensive RHI exposure: Hobey Baker sustained severe and innumerable hits to the head on the gridiron and the ice during his athletic career.
2. Progressive neurobehavioral change: Hobey had a significant and documented transition from a humble sportsman to an impulsive risk-taker with emotional volatility, risk-taking behavior, and apathy toward death.
3. Impaired judgment and risk assessment: Hobey performed reckless stunts and took an unnecessary final flight.
4. Lack of alternate neurological explanation: Baker had no documented history of substance misuse or other psychiatric diagnoses.

While a definitive CTE diagnosis is impossible without neuropathology, the clinical constellation does closely align with the modern TES criteria. However, attributing Baker’s behavioral deterioration and fatal decision-making to CTE-related neuropathology must be tempered by a frank acknowledgment of where the science currently stands. Traumatic encephalopathy syndrome was designed as a research construct, rather than a clinical diagnosis. The link between TES and confirmed CTE neuropathology, while plausible and supported by clinicopathological correlation studies, remains speculative at this time.

The Hobey Baker who boarded the repaired SPAD on that final flight may no longer have been capable of making the risk calculations the moment demanded. However, there are other possibilities that must be entertained to explain his decline. The broader differential diagnosis in Baker’s case, which would include post-concussive syndrome, major depression, bipolar disorder (with a depressive crash after the manic demands of combat), thrill-seeking personality disorder, genuine psychosocial weight of a broken engagement, and the post-athletic identity collapse, should be considered. Furthermore, it is possible that several of these conditions may have co-existed and amplified his potential underlying neuropathology. The overlap between post-concussive syndrome and early TES is particularly relevant here: both can produce an identical phenotype of mood instability, anhedonia, poor impulse control, and loss of future orientation that Baker displayed. Rarer conditions, such as early-onset Huntington’s disease or a leukodystrophy, would fit a picture of behavioral changes and mood disturbances in a young man, although the absence of documented motor features and the clear psychosocial precipitants make these less likely primary explanations. Finally, genetic susceptibility factors may also have influenced an individual’s response to RHI. In particular, the ApoE4 gene has been associated with less favorable neurological outcomes after brain injury and may increase vulnerability to trauma-related neurodegenerative processes (22). Baker’s genotype is unknown, but the presence of such a risk factor could have theoretically heightened his susceptibility to the cumulative effects of RHI.

## Conclusion

We are not arguing that Hobey Baker intentionally ended his life or that RHIs alone predetermined his death. Rather, we propose that the cumulative head trauma plausibly altered his emotional regulation, impulse control, and executive function—factors that may have contributed to his fatal decision-making on his final flight.

The frontal and limbic systems—regions susceptible to RHIs—govern judgment, inhibition, and risk assessment. Subtle impairments in these can manifest as heightened impulsivity and diminished hazard appraisal. In aviation, where survival depends on disciplined restraint, alterations can be catastrophic. Viewed using a contemporary lens, Baker’s life reads not as a heroic myth or tragic romance, but as a case study in the long-term consequences of unmitigated head trauma.

Hobey Baker remains immortalized as the archetype of the American amateur athlete—skilled, humble, and courageous. However, the same fearlessness that defined his athletic greatness exposed him to repeated head trauma in an era devoid of protective equipment and concussion awareness. Modern neuroscience suggests that repetitive head trauma can produce neurobehavioral changes including impulsivity, mood instability, apathy, and impaired judgment. Baker’s documented exposure to head trauma, personality evolution, and fatal decision-making align with these patterns.

The hypothesis that RHI may have contributed to Baker’s death is reasonable, historically grounded in what we now know about young collision athletes and worth taking seriously as a lens through which to understand his trajectory.

Whether his final flight was a tragic accident or self-inflicted harm may never be resolved. What can be asserted is that Hobey Baker’s story underscores an enduring lesson: the cumulative- and ultimately multiplicative—effects of repetitive head trauma may shape not only an athlete’s career, but also the arc of his or her life. Each injury lowers the threshold for the next, and the behavioral changes that follow place the athlete in ever-greater danger: this creates a cascade that becomes as difficult to escape as it is to recognize.

## Data Availability

The raw data supporting the conclusions of this article will be made available by the authors, without undue reservation.
